# Population Structure and Climate Effects on *Geckobia* Infestation in *Ptyodactylus* Geckos from Israel and West Bank, with Descriptions of *G. parva* sp. nov. and *G. inermis* sp. nov.

**DOI:** 10.3390/ani15233461

**Published:** 2025-11-30

**Authors:** Monika Fajfer-Jakubek, Bożena Sikora

**Affiliations:** 1 Department of Molecular Biology and Genetics, Institute of Biological Sciences, Cardinal Stefan Wyszynski University, Wóycickiego 1/3, 01-938 Warsaw, Poland; 2 Department of Animal Morphology, Faculty of Biology, Adam Mickiewicz University, Uniwersytetu Poznańskiego 6, 61-614 Poznań, Poland; boszka@amu.edu.pl

**Keywords:** Acari, scale mites, ectoparasites, species delimitation, *Ptyodactylus*, environmental filtering, climate gradient, museum specimens, prevalence, Middle East

## Abstract

Scale mites of the genus *Geckobia* (Pterygosomatidae) are highly specialized permanent parasites of geckos, but their diversity and ecology in arid environments remain poorly understood. We examined 1135 museum specimens of *Ptyodactylus* geckos collected from 1965 to 1991 across Israel and the West Bank’s Mediterranean–desert climate gradient to investigate environmental effects on *Geckobia* mite distributions and population structure. We analyzed prevalence, intensity, population structure, and seasonal patterns across three climate zones using standard parasitological methods and Köppen–Geiger climate classification. We describe two new species, *Geckobia inermis* sp. nov. and *G. parva* sp. nov., from *Ptyodactylus puiseuxi* and provide the first descriptions of previously unknown life stages: the male and nymphchrysalis of *G. squameum* and the imagochrysalis and larva of *G. bochkovi*. We report *P. oudrii* as a new host for *G. synthesys* and address taxonomic confusion regarding northern Israeli host populations following recent phylogenetic revisions of *Ptyodactylus.* Only 37 hosts were infected (3.26% prevalence), with a significant female bias in *G. squameum* populations. Most mites (94.6%) concentrated in the tympanum, where we documented a “double skin plug”, closing the ear opening and creating favorable microenvironments for mite survival. The results demonstrate climate as the primary factor structuring mite distributions: environmental filtering showed systematic prevalence decline from Mediterranean zones (4.3%) to desert-edge areas (1.1%), representing a 3.9-fold gradient that exceeded host species effects by 5.2-fold. Populations exhibited phenological plasticity, with Mediterranean mites peaking in winter versus spring activity in semi-arid zones. These findings reveal how climate constrains ectoparasite persistence in arid systems, with implications for understanding parasite responses to environmental change.

## Simple Summary

Scale mites of the genus *Geckobia* are parasitic mites that spend their entire life on gecko lizards, hiding in protected spots on the host’s body (e.g., beneath the scales, in axillas, and between claws or in ear cavities). These mites are highly specialized and typically found only on specific gecko species. We examined over 1100 preserved gecko specimens from an Israeli museum, collected between 1965 and 1991, to understand how environmental conditions affect these mite populations across the country’s climate gradient from Mediterranean coastal areas to desert regions. Only 37 geckos were infected, showing that these parasites are much rarer in dry environments compared to tropical regions, where similar mites can infect up to 100% of geckos. We discovered that mites were nearly four times more common in Mediterranean climate zones than in desert areas and that they show different seasonal activity patterns depending on local climate conditions. We also discovered two new mite species and documented a previously unknown “double skin plug” that blocks gecko ear openings, creating humid conditions that help mites survive in desert climates. This research helps us understand how climate change might affect these parasite–host relationships and provides important baseline data for future studies of ecosystem changes in arid regions.

## 1. Introduction

Scale mites of the family Pterygosomatidae Oudemans, 1910 (Acariformes: Prostigmata) are among the most abundant and highly specialized permanent ectoparasites of reptiles [1]. The only exceptions are species of the genus *Pimeliaphilus* Trägårdh, 1905, which are associated not only with lizards but also with terrestrial arthropods [2] and a single species known only from the holotype, *Bharatoliaphilus punjabensis* Prasad, 1975, found on a dove *Streptopelia decaocto* (Frivaldszky) [3]. Currently, the family comprises over 180 described species distributed across all zoogeographical regions except for Antarctica [4].

Among Pterygosomatidae, the genus *Geckobia* Mégnin, 1878 is the most diverse and species-rich, comprising over 80 described species and subspecies associated mostly with lizards from tropical and subtropical regions worldwide [1,5]. The genus exhibits remarkable host specificity, with most species being mono- or oligoxenous parasites typically restricted to single gecko species or closely related species [6,7]. This high degree of host specificity suggests extensive coevolutionary relationships and indicates that actual diversity may be substantially underestimated, particularly in regions with high gecko endemism [5,7]. These obligate parasites have evolved remarkable morphological adaptations for living on the host’s body, including dorsoventrally flattened bodies that help them hide beneath the scales, anteriorly directed legs for secure attachment, and specialized chaetotaxy patterns that enhance host gripping [1,8].

Currently, *Geckobia* species are arranged into six species groups based on trochanter-tibia chaetotaxy patterns of legs I–IV (*latasti*, *haplodactyli*, *ovambica*, *indica*, *nitidus*, and *simplex* groups), with additional subdivision into groups A and B based on tarsal chaetotaxy differences in leg I [9,10]. However, approximately one-third of described species remain unassigned to any group due to unique morphological characteristics or inadequate original descriptions, highlighting ongoing taxonomic challenges within this morphologically diverse genus [6].

The Mediterranean–desert transition zone of the Levant provides an ideal model system for examining how climate constrains ectoparasite occurrence. Environmental filtering—the process by which abiotic conditions exclude species from habitats where environmental extremes exceed their physiological tolerances—is a fundamental mechanism structuring species distributions along climate gradients [11]. This filtering is predicted to be particularly strong for arthropod parasites in arid environments, where desiccation stress limits survival and reproduction [12,13]. Israel encompasses a dramatic climatic gradient from Mediterranean conditions (>600 mm annual rainfall) through semi-arid zones (200–400 mm annual rainfall) to hyper-arid desert environments (<50 mm rainfall), compressed within a remarkably small geographic area [14]. This steep environmental gradient creates predictable variation in temperature, humidity, and precipitation that fundamentally constrains arthropod physiology and survival, particularly for ectoparasites vulnerable to desiccation stress. Unlike free-living arthropods that can seek favorable microhabitats, permanent ectoparasites are restricted by both their host’s distribution and the conditions available on the host’s body surface. In tropical systems, pterygosomatid mites achieve high prevalence rates reaching 100% and exploit diverse body regions as microhabitats to avoid interspecific competition [15]. Whether similar patterns occur in arid-adapted populations or if environmental stress fundamentally alters pterygosomatid ecology remains largely unexplored.

Recent regional surveys have documented *Geckobia* diversity from adjacent Eastern Mediterranean areas. Bertrand et al. [16] described *Geckobia estherae* Bertrand, Pflieger, and Sciberras, 2012 from Maltese *Tarentola mauritanica* (Linnaeus, 1758) and reviewed Mediterranean *Geckobia* species with specialized scale-like ventral setae. Bertrand et al. [17] reported *G. sharygini* Bertrand, Kukushkin, and Pogrebnyak, 2013 from Crimean populations of *Mediodactylus danilewskii* (Strauch, 1887)—a Mediterranean gecko reaching its climatic limits under continental conditions—while Eren and Açıcı [18] documented *G. turkestana* Hirst, 1926 from northeastern Anatolia on *Mediodactylus* cf. *kotschyi* (Steindachner, 1870). Notably, Bertrand et al. [17] observed reduced pterygosomatid prevalence in this northernmost marginal population, experiencing cold winters and temperature extremes, while Bertrand et al. [16] similarly noted declining prevalence toward desert margins in North African *Tarentola* populations. These observations suggest that climate imposes physiological constraints on mite survival at distributional extremes. However, quantitative analyses across complete environmental gradients—from climatically favorable Mediterranean refugia through semi-arid transitions to physiologically limiting hyper-arid deserts—remain lacking for pterygosomatid mites, particularly for Phyllodactylidae-associated species in this biogeographic transition zone.

Fan-footed geckos of the genus *Ptyodactylus* Goldfuss, 1820 (Squamata: Phyllodactylidae) are among the most successful reptilian colonizers of rocky habitats across North Africa and the Middle East. Recent molecular phylogenetic studies have revealed considerable cryptic diversity within this genus, with multiple lineages showing strong geographic structure and ecological specialization [19]. In Israel and the West Bank, three species dominate in different ecological zones: *P. guttatus* Heyden, 1827 in the Negev and southern regions; *P. puiseuxi* Boutan, 1893 in northern Mediterranean areas and parts of the West Bank; and *P. hasselquistii* Donndorff, 1798 with distributions primarily in the Jordan Valley and coastal plains [20]. Previous studies on *Geckobia* parasitizing Israeli *Ptyodactylus* have been limited to taxonomic descriptions of species. Bertrand et al. [21] described *G. squameum* Bertrand; Paperna; and Finkelman, 1999 from *P. guttatus*, followed by Fajfer [22], who described *G. bochkovi* and *G. synthesys* from the same host. However, these studies provided no ecological context regarding how environmental conditions structure mite populations across the region’s dramatic climate gradient.

Museum collections represent invaluable resources for temporal ecological analysis, particularly for specialized parasites with naturally low detection probabilities and patchy distributions. For scale mites of the family Pterygosomatidae, preservation effects on detectability are expected to be minimal because the mites remain firmly attached to specific hidden microhabitats on the host’s body [23], which provide protection from mechanical detachment during specimen handling and storage. While direct museum versus field comparisons for Pterygosomatidae are scarce, analogous studies on temporal ectoparasites of reptiles, trombiculid mites, have demonstrated that large-scale museum-based surveys (on 2425 museum host specimens from 77 Phrynosomatidae species) yield prevalence patterns closely resembling those from field studies, supporting the reliability of museum collections for trend analyses [24].

To our knowledge, no comparable large-scale ecological studies of Pterygosomatidae across Mediterranean–desert gradients have been published. Most pterygosomatid research focuses on tropical or subtropical systems with high infection rates (28–100% prevalence) [15,25,26], while systematic surveys of museum collections for reptile ectoparasites [24] reveal no analogous datasets examining over 1000 hosts across climate gradients in arid regions. This gap is particularly significant given that arid environments represent fundamentally different selective pressures for permanent ectoparasites compared to humid tropical systems, where most Pterygosomatidae research has focused [1,4,5].

Here, we present the first comprehensive ecological analysis of *Geckobia* mites associated with *Ptyodactylus* geckos in Israel and the West Bank, based on examination of 1135 museum specimens collected from 1965 to 1991 across multiple biogeographic districts. We describe two new species, *Geckobia parva* sp. nov. and *G. inermis* sp. nov., both from *Ptyodactylus puiseuxi*, expanding knowledge of Middle Eastern pterygosomatid diversity. Additionally, we provide the first descriptions of previously unknown life stages: the male and nymphchrysalis of *G. squameum* and the imagochrysalis and larva of *G. bochkovi*. We report *Ptyodactylus oudrii* as a new host for *G. synthesys* and address taxonomic confusion regarding northern Israeli *Geckobia* populations through host re-identification based on recent *Ptyodactylus* phylogenetic revisions. We analyze the prevalence, intensity, and abundance of infections, revealing that only 37 of 1135 hosts (3.26%) were parasitized, with significant female-biased sex ratios in several species and distinct microhabitat preferences (94.6% of mites in the tympanic cavity). We test whether environmental filtering along Israel and the West Bank’s Mediterranean–desert gradient structures mite distributions more strongly than host species identity, demonstrating a 3.9-fold decline in prevalence from Mediterranean to desert-edge zones. Finally, we examine seasonal phenological patterns across climate zones, revealing adaptive temporal shifts, with Mediterranean populations peaking in winter while semiarid populations shift activity to spring, suggesting plasticity in response to local climatic constraints.

## 2. Materials and Methods

### 2.1. Specimen Collection and Morphological Analysis

All available *Ptyodactylus* specimens (n = 1135) stored in jars containing 75% ethyl alcohol in the herpetological collection of the National Natural History Collections of the Hebrew University of Jerusalem (HUJ) were examined for the presence of mites under a Nikon SMZ745 stereomicroscope (Nikon Instruments Inc., Melville, NY, USA) (Supplementary Table S1). Mites collected from different regions of the host’s body were counted to assess their site preferences, then transferred to small 2 mL vials filled with 75% ethyl alcohol. After each lizard was inspected, the remaining ethyl alcohol from the jar bottom was poured onto Petri dishes and examined under the stereomicroscope to search for any detached mites. Only one host record (0.09% of all examined, 2.7% of infected hosts) contained detached mites noted as “jar” for body location in Supplementary Table S1. This specimen was included in prevalence and species identification analyses but excluded from microhabitat analyses due to the unknown original location of the host’s body. Data for all checked hosts (catalog numbers) was recorded directly from jar labels, and then it was used to retrieve associated host metadata from the Global Biodiversity Information Facility [27].

Prior to mounting in Hoyer’s medium, mite specimens were cleared and softened in Nesbitt’s solution at +45 °C for a period of 8 to 24 h. All specimens were mounted as vouchers using a modified Faure’s Berlese medium on glass slides using standard protocols described by Krantz and Walter [28]. All microscopic images were captured using a Leica DMD108 microscope (Leica Microsystems, Wetzlar, HE, Germany). In species descriptions, names of leg and idiosomal setae followed Grandjean’s nomenclature [29,30] as described by Norton [31], whereas those of palpal setae followed Grandjean’s later work [32]. Comparative morphological data for the described *Geckobia* species from Mediterranean–Middle Eastern geckos are provided in Supplementary Table S2. All measurements and scale bars in all figures are presented in micrometers (μm). Scientific names of lizards follow Uets et al. [33].

All examined specimens and prepared vouchers were deposited in the arachnid collection of the HUJ and in the Department of Molecular Biology and Genetics, Institute of Biological Sciences, Cardinal Stefan Wyszynski University in Warsaw (CSWU), Poland.

Of a total of 1135 *Ptyodactylus* specimens, 1010 had sufficient locality data for Köppen–Geiger climate classification [34] and were included in the main ecological analyses. The remaining 125 specimens lacked precise geographic information and were excluded from the climate-based analyses but retained for species descriptions and overall prevalence calculations.

### 2.2. Parasitological Data Analysis

Basic parasitological parameters (prevalence, intensity, abundance) were calculated following [35]. Each host specimen was identified by its unique catalog number from HUJ, and all analyses were conducted at the host level. All life stages were summed to determine the total mite load per host. Parameters were calculated both overall for all mite species combined and separately for each mite species. All mean values are presented with standard deviations (mean ± SD) and medians with interquartile ranges (IQR). For continuous-variable contrasts (e.g., intensity across hosts), we report standardized mean differences as Cohen’s d with Hedges’ small-sample correction (g) and 95% confidence intervals.

### 2.3. Population Structure and Sex Ratio Analysis

For each *Geckobia* species, we analyzed the proportion of different life stages (adults: females + males; juveniles: larvae + deutonymphs + protonymphs; chrysalids: imagochrysalis + nymphchrysalis) to assess population structure and reproductive patterns at the species level. Throughout this study, “population” refers to all individuals of a given *Geckobia* mite species pooled across all examined gecko hosts within a specified spatial unit (climate zone or district), not to mites on individual host specimens. Sex ratios were calculated as males per female at the species level, summing all adult specimens of each Geckobia species across all hosts and climate zones, to test for systematic sex ratio biases characteristic of each mite species. Reproductive patterns were inferred from temporal variation in juvenile (larvae + nymphs) proportions and from seasonal peaks in juvenile abundance, which indicate active reproduction periods. Exact binomial tests were performed to test whether observed sex ratios deviated significantly from the expected 1:1 ratio for each *Geckobia* species, using the formula *p* = 0.5 under the null hypothesis of equal sex distribution. Tests were conducted two-sided with α = 0.05. Holm step-down correction was applied to control the family-wise error rate for multiple comparisons across species.

### 2.4. Köppen–Geiger Climate Classification and Spatial Patterns

Climate zone assignment using the Köppen–Geiger system [34] successfully classified 748 hosts (74.1% of total), with the remaining 262 hosts (25.9%) assigned to simplified categories based on administrative boundaries. For the purposes of this analysis, the data were filtered to include only records from Israel and the West Bank (n = 1010), defined geographically by a bounding box (29.0–33.6° N, 34.2–35.9° E) and administratively by district or country labels (e.g., “northern district”, “central district”, “Israel”). For ecological analysis, these were aggregated into broader categories: Mediterranean: Csa (hot-summer) and Csb (warm-summer), 628 hosts; semi-arid (BSh, 288 hosts); desert-edge (BWh, 94 hosts); and other (representing 86 hosts with insufficient climatic data). When GPS was unavailable, climate followed district/locality obtained from jar labels; unresolved cases were labeled “other” and excluded from three-zone analyses.

Analyses were conducted both by climate zone (to test environmental filtering) and by host species (to test host specificity effects). Sampling years (1965–1991) were not analyzed separately because (1) our hypotheses test spatial (climate zone) patterns, not temporal trends; (2) annual sample sizes were insufficient for statistical inference (0–7 infected hosts per year based on collection records); and (3) Israel’s major climate zone boundaries (Köppen–Geiger classification) remained stable throughout this period, with significant shifts occurring only in recent decades [14,36,37]. To assess environmental filtering across the climate gradient, we tested for systematic changes in mite prevalence across Köppen–Geiger climate zones using the final dataset of 1010 hosts from Israel and having full data. Environmental filtering strength was quantified as the fold-change in prevalence between Mediterranean and desert-edge zones. Distribution maps were created to visualize mite occurrence patterns across host ranges and climate zones. Geographic range utilization was assessed by determining the proportion of climate zones where mites occurred relative to zones where hosts were present. To test the relative importance of climate versus host species in predicting mite occurrence, we compared predictive power using chi-square tests and Cramér’s V as effect size measures.

For seasonality analysis, we included all hosts with recorded collection month (N = 970 of 1135 total), representing all four gecko species (*P. guttatus*, *P. puiseuxi*, *P. hasselquistii*, *P. oudrii*). The remaining 165 hosts lacked monthly data and were excluded from seasonal analyses only. Collection dates were grouped into seasons following Israeli climate patterns: winter (December–February), spring (March–May), summer (June–August), and autumn (September–November). Seasonal sampling effort varied across climate zones: Mediterranean (619 hosts total), semi-arid (277 hosts), and desert-edge (74 hosts). Phenological plasticity was assessed by comparing seasonal infection patterns across climate zones following Israeli climate patterns as defined by Goldreich [14]. Peak reproductive periods were identified as months with the highest infection rates within each climate zone. Phenological shifts were quantified as the temporal difference in peak activity between climate zones. To distinguish between true biological patterns and sampling artifacts, we explicitly quantified sampling effort (number of hosts examined) versus detection success (number infected) for each climate–season combination.

### 2.5. Statistical Analysis

All statistical analyses were conducted using Python 3.9 with scipy.stats library, visualizations with the matplotlib library. For contingency table analyses, Fisher’s exact test was used instead of chi-square when >20% of cells had expected values < 5.

### 2.6. Statistical Power and Effect Size Analysis

Power analysis was conducted post hoc using G*Power 3.1.9.7. For the main climate gradient effect (Mediterranean vs. semi-arid vs. desert-edge), with 1010 hosts and 37 infections distributed non-uniformly across zones, we achieved approximately 60% power to detect the observed effect (Cramér’s V = 0.078, 95% CI: 0.045–0.112) at α = 0.05. For species-level analyses, sample sizes limited statistical power, *G. parva* (n = 4 hosts, power < 20%) and *G. inermis* (n = 1 host, power insufficient), necessitating descriptive rather than inferential statistics for these species. Sex ratio analyses achieved 80% power only for species with ≥40 adult specimens (*G*. *bochkovi*, *G. squameum*). All effect sizes are reported with 95% confidence intervals calculated using bias-corrected bootstrap (n = 10,000 iterations) for non-normally distributed ecological count data. Cohen’s d for continuous variables, Cramér’s V for categorical associations, and risk ratios (RRs) for prevalence comparisons are provided throughout.

## 3. Results

### 3.1. Description

Systematics

Family: Pterygosomatidae Oudemans, 1910

Genus: *Geckobia* Mégnin, 1878

Species group *latasti* sensu Fajfer [5] (Jack’s group I [9])

*Geckobia inermis* sp. nov.

**Figure 1:**
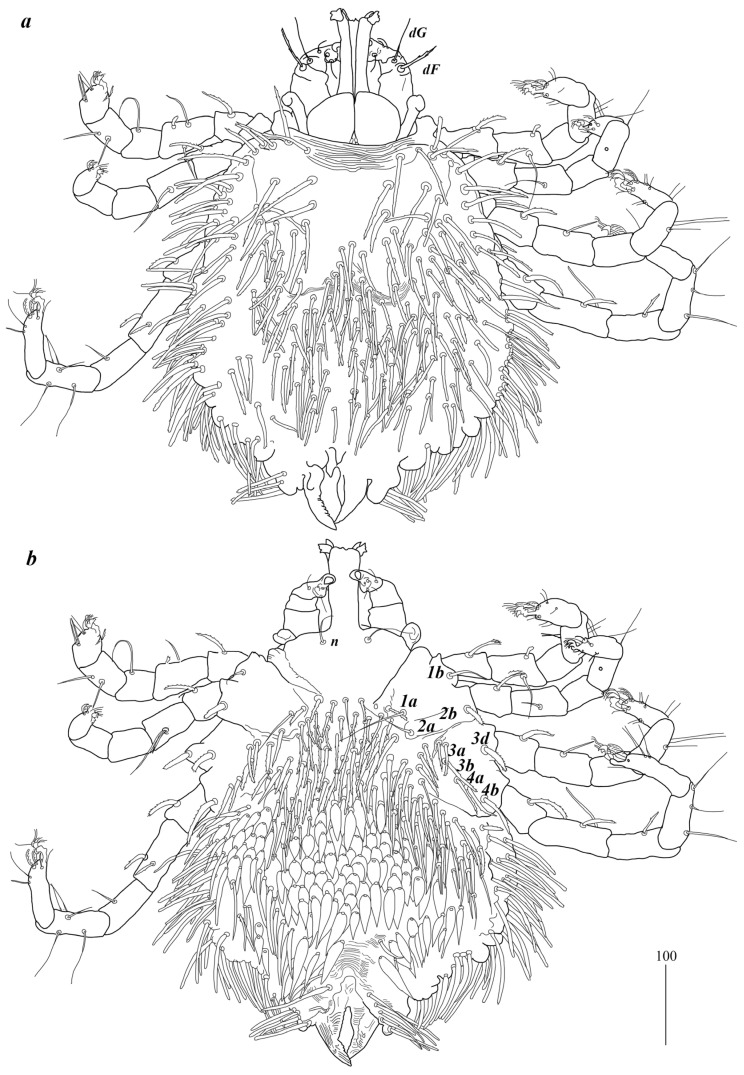
*Geckobia inermis* sp. nov, female: (**a**) in dorsal view; (**b**) in ventral view.

Description. Female (holotype, range for paratype) (Figure 1). *Gnathosoma*. Chelicerae 185 (220) long. Swollen, proximal part of cheliceral base 80 (110) long and slender distal part 105 (110) long. Movable cheliceral digit three-pronged, fixed cheliceral digit spinous, and approximately 15 (15) long. Palpal femur with filiform smooth or with barely discernible serration seta *dF* 80 (40) long; palpal genu with filiform smooth seta *dG*, 85 (80) long. Palpal tibia with three smooth setae (*dTi*, *l’Ti* and *l”Ti*) and slender curved claw. Palpal tarsi with four smooth setae. Subcapitular seta *n* filiform and smooth, 70 (70) long. Each branch of peritremes with barely visible chambers 100 (135) long. Hypostome with three-pronged apex. *Idiosoma* 400 (420) wide long and 460 (450) long. Dorsum (Figure 1). Propodonotal shield smooth and well outlined, slightly concave in anterior and posterior part, 250 (255) wide and 85 (110) long in middle part. On propodonotal shield 13 (13) pairs of very slightly plumose thick and blunt-pointed setae, 50–70 long. One seta situated antero-laterally shorter, about 25 (30) long. Posteriorly and laterally to propodonotal shield, numerous setae resembling setae situated on propodonotal shield, 50–75 long. Eyes absent.

Venter. Anterior part with 1–2 rows of filiform smooth setae, about 30 long, below 4 rows of slightly plumose thicker and tapered setae, 35–40 long. Anteromedial part of idiosoma with plumose setae. Posterior half of idiosoma with lanceolate setae. These setae about 40 (40) long and 15 (15) wide. Most posterior peripheral setae more elongated and narrower than setae in medial part (50 long and 10 wide). Genital region. Genital setae represented by four pairs of slender blunt pointed setae *g1–g4.* Setae *g1* and *g2* about 20 long, *g3* about 10 long, and *g4* about 30 long. Pseudanal series represented by 11 pairs of blunt-pointed smooth and flattened setae *ps1*–*ps11*, about 50 (40–55) long. *Legs*. Coxal setation: *1a*, *1b*, *2a*, *2b*, *3a*, *3b*, *3c*, *3d*, *4a*, *4b* and *4c* arranged in formula: 2–2–4–3. Setae *1a*, *1b*, filiform and smooth; *2a* and *3d* serrate; *2b* thick, short and plumose; *3a*, *3b*, *3c*, *4a*, *4b* thick, slightly plumose, tapered and resembling those on venter. Two plumose setae present between coxal plates I and II. Leg chaetotaxy as follows: tibiae I–IV (5–5–5–5), genua I–IV (1–0–0–1), femora I–IV (3–2–2–2), and trochanters I–IV (1–1–1–1). Setae *dTiI–IV*, *ld’TiI*, *ld”TiIV*, *v’TiI–IV*, *v”TiI–IV*, *ldGI*, *ld GIV, dl”FI–FIV*, *vFI–IV* filiform and smooth; *vTrI-IV*, *dl’FI* serrate. Setation of tarsi I: 14 setae (*ft*, *tc*’, *tc*”, *p*’, *p*”, *a*’, a”, *it*’, *it*”, *u*’, *u*”, *vs*’, *vs*”, and *pl*’) and solenidion *w*1; tarsi II: 10 setae (*tc*’, *tc*”, *p*’, *p*”, *a*’, a”, *u*’, *u*”, *vs*’, and *vs*”) and *w*1; tarsi III and IV with 10 setae each (*tc*’, *tc*”, *p*’, *p*”, *a*’, *a*”, *u*’, *u*”, *vs*’, and *vs*”). Solenidion *w1* (about 25 long) longer than seta *ft* (about 5 long). Setae *tc*’, *tc*”, *it*’, and *it*” of leg I represented by euphatidia; *tc*’ and *tc*” of legs II–IV, *u*’, *u*”, *vs*’, *vs*”, *a*’, *a*” and *pl*’ of legs I–IV filiform.

**Figure 2:**
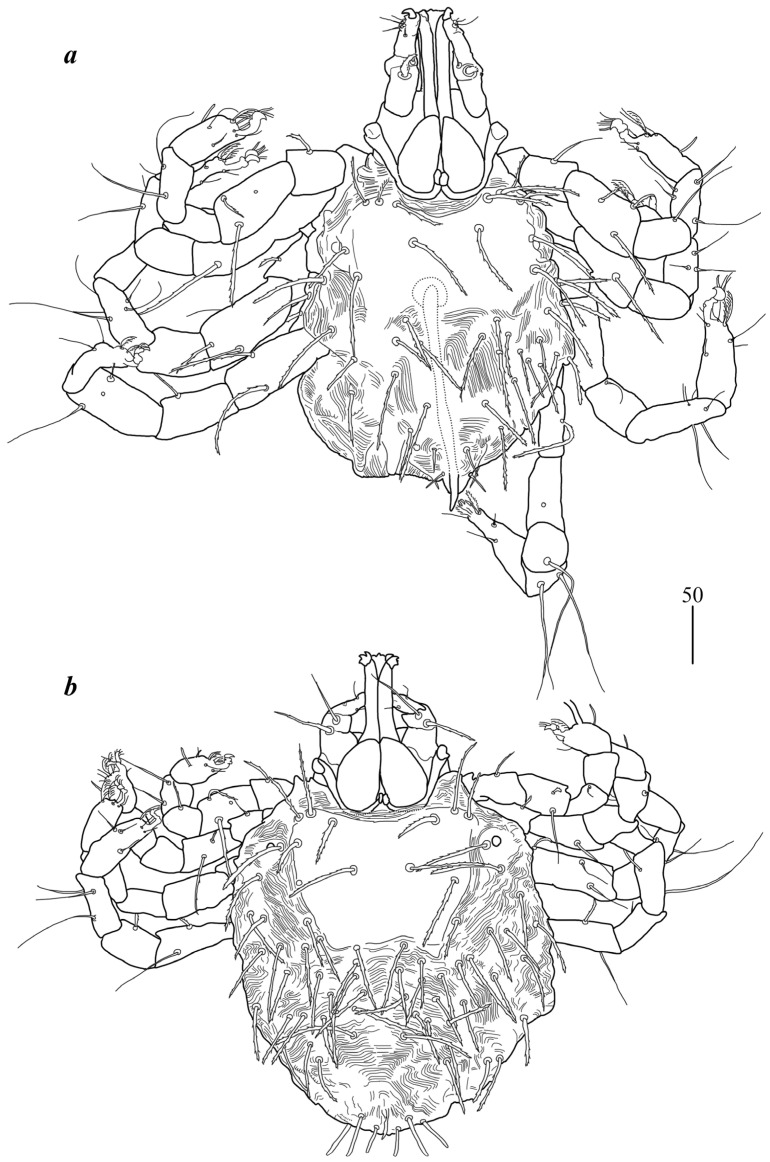
*Geckobia inermis* sp. nov.: (**a**) male in dorsal view; (**b**) deutonymph in dorsal view.

Male (Figure 2a). *Gnathosoma* as in female. Chelicerae about 145 long; slender cheliceral part 80 long, swollen basal part 65 long. Fixed cheliceral digit about 10 long. Setae *dF* thick, serrate, about 20 long; setae *dG* filiform, smooth, about 50 long. Subcapitular seta *n* 35–40 long. Each branch of peritremes about 100 long. Hypostome with ornamented apex. *Idiosoma* 215 (200–255) wide, 265 (260–295) long. Dorsum with propodonotal shield 80 long, 155 wide, accompanied by ocular plate on lateral margins. On propodonotal shield 7 pairs of serrate setae: 4 pairs situated antero-laterally (30–60 long), 1 pair medially (60 long), 2 pairs postero-laterally (including 1 on ocular plate), and 3 pairs of longer setae 35–40 long. Medial and posterior part with about 22 pairs of serrate setae, 30–50 long. Aedeagus 185 long. Genital cone with 2 filiform setae 35 and 10 long situated dorsally, and one filiform seta situated ventrally, 25 long. Venter with 5 pairs of setae (about 45 long) situated medially. *Legs.* Coxae in formula: 2–2–2–2. Setae *1a*, *1b*, *2a*, *2b* filiform and smooth; setae *3a*, *3b*, *4a* and *4b* serrate. Setae of tibiae–trochanters I–IV as in female.

Deutonymph (Figure 2b). *Gnathosoma* as in female. Chelicerae 110–130 long; swollen cheliceral part and slender distal part about 60 long. Fixed cheliceral digit 10 long. Setae *dF* slightly serrate, 60 long; setae *dG* filiform, smooth, 50 long. Subcapitular setae *n* about 45 long. Each branch of peritremes about 90 long. *Idiosoma* 250–275 long and 175–234 wide. Dorsum. Propodonotal shield about 90 long and 140 wide, with 5–6 pairs of slightly serrate setae: 3 pairs situated antero-laterally (35–60 long), 1 pair medially (55–60 long), 1 pair medio-laterally (about 60 long). Laterally to propodonotal shield an eye on oval ocular plate (20 wide and 25 long) with associated serrate seta (60 long). About 25 pairs of serrate setae (35–60 long) situated in lateral and medial part of idiosoma. Venter with 12–13 shorter serrate setae (15–25 long) in antero-medial part, and 25 longer setae (35–65 long) in medial part. Coxae in formula: 2–2–2–2. Setae *1a*, *1b* filiform; setae *2a*, *2b*, *3a*, *3b*, *4a*, *4b* serrate. *Legs.* Setae of trochanters-tarsi I–IV as in female. Genital area with 2 pseudanal setae *ps1* and *ps2* with barely visible serration and fine-pointed setae *g1–g3*. Setae *ps1* 30–35 long, setae *ps2* 25 long; setae *g1–g3* about 15 long.

**Figure 3:**
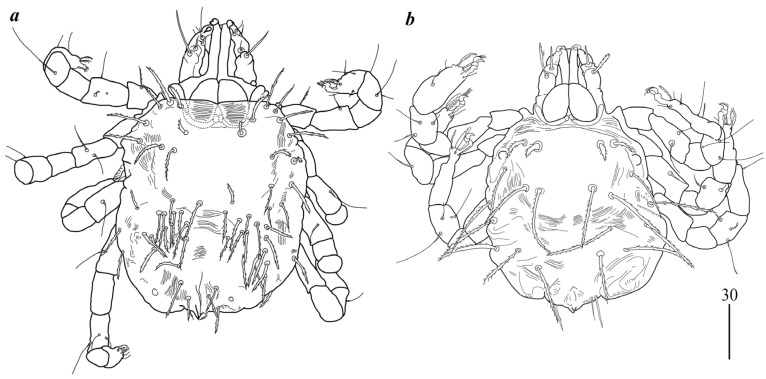
*Geckobia inermis* sp. nov.: (**a**) protonymph; (**b**) larva in dorsal view.

Protonymph (Figure 3a). *Gnathosoma* as in female. Swollen cheliceral part 35 long, slender distal part 30 long. Setae *dF* slightly serrate and 55 long, setae *dG* smooth and 55–60 long. Each branch of peritremes 65 long. *Idiosoma* 210–220 long and 190–200 wide. Dorsum with densely serrate setae. Propodonotal shield 125 wide and 95 long with 4 setae present on shield: 2 short setae situated anteriorly (about 25 long) and 2 longer setae situated medially (60 long). Laterally to propodonotal shield eye on ocular plate present with associated serrated seta (65 long). In medial part numerous setae, 35–45 long, present. Venter with tapered setae 35–40 long. In anterior part about 6 short setae (20–25 long), in medial part numerous longer setae (30–40 long). Genital setae *g1* with barely discernible serration, *g2–g3* smooth, setae *g1*–*g3* 15–20 long. Pseudanal setae *ps1* and *ps2* slightly serrate and 30–35 long. *Legs* as in female.

**Figure 4:**
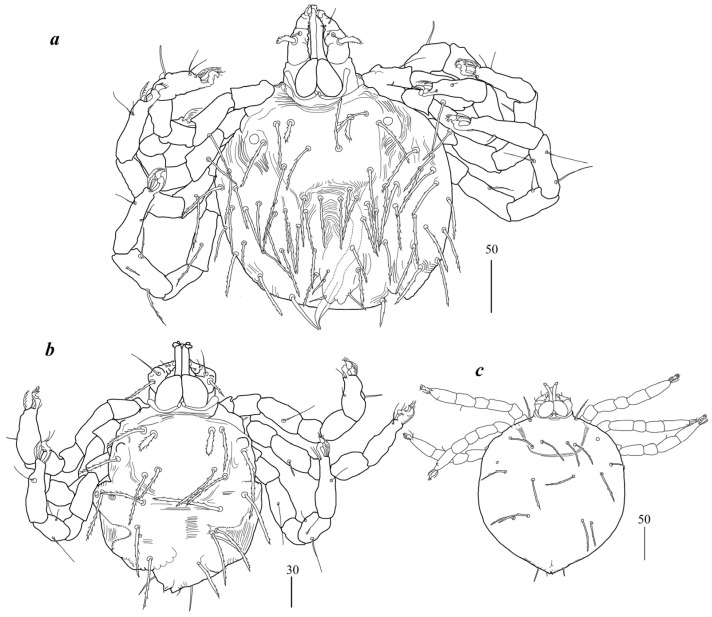
(**a**) *Geckobia squameum*, male in dorsal view; (**b**) *Geckobia bochkovi*, larva in dorsal view; (**c**) *Geckobia parva*, larva in dorsal view.

**Figure 5:**
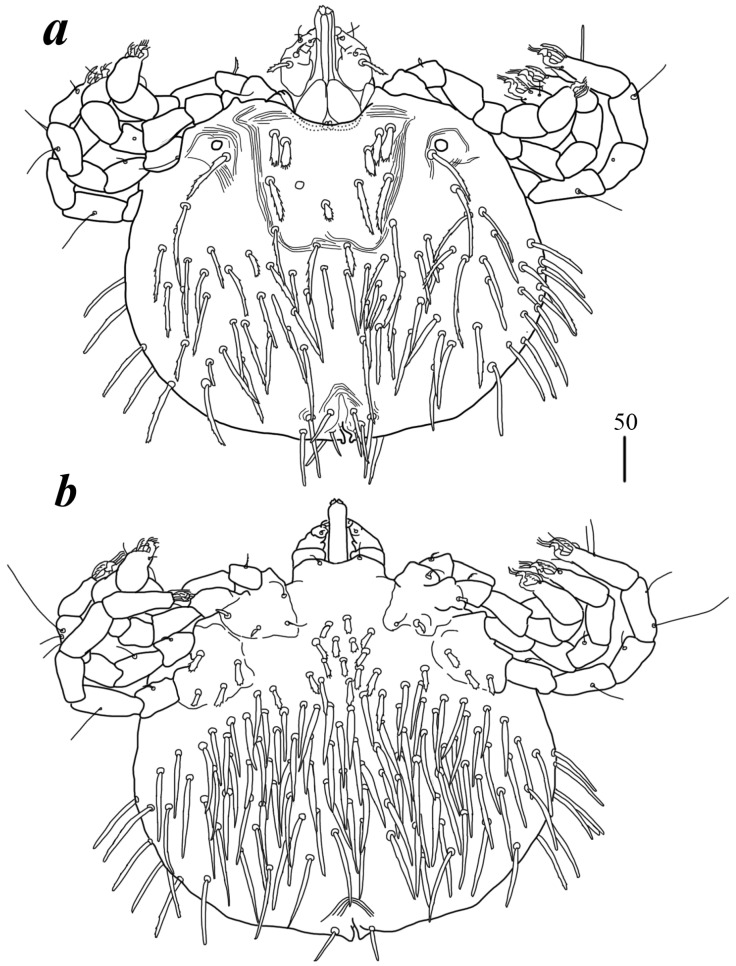
*Geckobia parva* sp. nov., female: (**a**) in dorsal view; (**b**) in ventral view.

Larva (Figure 4a). *Gnathosoma* as in female. Chelicerae about 55–60 long; slender cheliceral part 30–40 long, swollen distal part about 30–35 long. Setae *dF* 25 long, setae *dG* 30 long. Peritremes about 30 long. Subcapitular setae *n* absent. *Idiosoma* 125–230 long and 115–220 wide. Dorsum. Propodonotal shield 65 long, 80 wide; bearing one pair of short densely serrate setae, 15 long, and three pairs of longer serrate setae, 35 long. Laterally to propodonotal shield eyes on oval ocular plates (15 wide, 25 long) with one associated serrate seta (35 long) present. Posteriorly to propodonotal shield six pairs of serrate setae, about 35–45 long. Venter devoid of any setation. Genital area with three filiform genital setae *g1–g3*, about 10 long, and two slightly serrate pseudanal setae *ps1–ps2* 20 long. Coxae in formula: 2–0–1. Setae *1a*, *1b* filiform; *3a* short, densely serrate. Setation of trochanters-tarsi I–III as in female and typical for pterygosomatid larva (Figure 5 and Table 2 in [23]).

Differential diagnosis. This new species is most similar to *Geckobia bochkovi* Fajfer, 2023, described from *Ptyodactylus guttatus* Heyden (Phyllodactylidae) in Israel [22]. Both species share the presence of a well-defined propodonotal shield, slightly serrate dorsal setae, lanceolate ventral setae, and a comparable leg chaetotaxy. However, the new species differs from *G. bochkovi* in several key morphological traits: the propodonotal shield lacks anterior and posterior concavity; the dorsal setae are uniform in size; the anterior part of the ventral surface bears 1–2 rows of filiform smooth setae; seta *2a* is serrate; coxal setae *3c* and *3d* are present; pseudanal series are represented by 11 pairs of blunt-pointed, smooth, flattened setae *ps1–ps11* and eyes are absent. In contrast, in *G. bochkovi*, the propodonotal shield is concave both anteriorly and posteriorly; the dorsal setae are fine-pointed and slightly increase in length posteriorly; coxal seta *2a* is smooth and filiform; coxal setae *3c* and *3d* are absent; setae *ps1*–*ps12* are slightly lanceolate with minute serration and tapered at tips, and eyes are present.

Type material. Holotype female and 1 female paratype, 2 males, 2 nymphs, 4 larvae from *Ptyodactylus puiseuxi* Boutan, 1893 (tympanum) (HUJ no. 18522), Israel: Northern District: Golan: Nahal, 6 May 1987, coll. Wered Werner.

Type material deposition. Holotype female, male, deutonymph and protonymph, 2 larvae in the HUJ (HUJINV-Acari_Pte00003.1–7), female paratype and 2 larvae in the CSWU (CSWU–Pte0019.1–3).

Etymology. This species name is derived from the name *inermis*, which is a Latin adjective meaning “unarmed” or “without spines”, referring to the blunt-pointed, smooth pseudanal setae of this species.

*Geckobia squameum* Bertrand, Finkelman, and Paperna, 2000

*Geckobia squameum* Bertrand, Finkelman, and Paperna, 2000: 294, figs 42−47

Male (Figure 4a). *Gnathosoma* as in female. Chelicerae 70 long; slender cheliceral part about 40 long, swollen basal part 30 long. Fixed cheliceral digit about 5 long. Setae *dF* thick, serrate, about 15 long; setae *dG* filiform, smooth, about 35 long. Subcapitular seta *n* 35 long. Each branch of peritremes about 45 long. Hypostome with ornamented apex. *Idiosoma* 175–190 long, 175–195 wide. Dorsum with propodonotal shield 85 long and 175 wide, accompanied by ocular plate on lateral margins. On propodonotal shield 5 pairs of serrate setae: 2 pairs situated antero-laterally (one seta shorter about 25 long, second setae about 50 long, 1 pair medially (65 long), 1 pairs postero-laterally, about 60 long, and one pair 60 long, near ocular plate. Medial and posterior part with about 23 pairs of serrate setae, 40–60 long. Aedeagus 110 long. Genital cone with one pair of setae about 25 long. Genital cone situated dorsally. Venter with 10 pairs of very slightly serrate setae (20–30 long) situated antero-medially and about 30 pairs of setae situated in posterior half of idiosoma. *Legs.* Coxae in formula: 2–2–2–2. Setae *1a*, *1b*, *2a*, *2b* filiform and smooth; setae *3a*, *3b*, *4a* and *4b* serrate. Setae of tibiae–trochanters I–IV as in female.

Nymph chrysalis. *Gnathosoma* with visible peritremes. *Idiosoma* almost circular (290 wide and 280 long in one specimen, 205 long and 230 wide in second) with fully formed male inside. Dorsal and ventral side of idiosoma devoid of any setation, only coxae visible.

Non-type material. Two males from *Ptyodactylus guttatus* Heyden, 1827 from Israel, coll. unknown; 6 males and 2 nymph chrysalis from same host, Israel, Northern District: 1 km South West of Tubas, 01.02.1986, coll. Yaacov Pesach.

Material deposition. Eight males and 2 nymph chrysalis in the CSWU (CSWU-Pte0017.1–10).

*Geckobia bochkovi* Fajfer, 2023

Geckobia bochkovi Fajfer, 2023: 252, figs 1−3

Imagochrysalis. *Gnathosoma* barely discernible, inserted at ventral surface of idiosoma. Peritremes with barely discernible chambers. *Idiosoma* 420–545 long and 375–490 wide. Only coxae I and II visible. Inside fully formed female visible.

Larva (Figure 4b). *Gnathosoma* as in female. Chelicerae about 60 long; slender cheliceral part 30 long, swollen part about 30 long. Setae *dF* filiform and slightly serrate, 25 long; setae *dG* filiform and smooth, about 35 long. Peritremes about 35 long. Subcapitular setae *n* absent. *Idiosoma* 130 long and 155 wide. Dorsum. Propodonotal shield 60 long and 75 wide; well-outlined, bearing four pairs of densely serrate setae: 2 pairs situated antero-laterally (one pair longer, 40 long, one pair shorter, 20 long), and 2 pairs situated posteriorly on the shield, both about 45 long. Laterally to propodonotal shield eye on ocular plate and one seta present (40 long). In posterior half of idiosoma five pairs of posterior setae 40–50 long present. Venter devoid of any setation. Genital area with 3 filiform genital setae *g1–g3*, 5–10 long, and two slightly serrate pseudanal setae *ps1–ps2* 15–20 and 20–25 long. Coxae in formula: 2–0–1. Setae *1a*, *1b* filiform; *3a* short, densely serrate. Setation of trochanters-tarsi I–IV as in female, except for presence in several specimens of *dGI*; setation of legs I–III typical for larva (Figure 5 and Table 2 in [30]).

Non-type material. One imago chrysalis from *Ptyodactylus guttatus* Heyden (HUJ no. 2915) (tympanum) from Israel, Haifa district, Coastal Plain: Atlit, April 1955, coll. Michael Warburg; 1 imago chrysalis from same host species (HUJ no. 2916) (tympanum) and locality, April 1955, coll. Michael Warburg; 1 larva with from same host (HUJ no 2798) (tympanum), Israel, Haifa district: Mount Carmel above Nesher, 15.02.1955, coll. Yehudah L. Werner.

Material deposition. Two imagochrysalis, 1 larva in CSWU (CSWU-Pte0018.3–6).

Remarks. Northern Israeli specimens require host verification through DNA barcoding (see Discussion in Section 4).

Species group *diversipilis* (sensu Jack [9])

*Geckobia parva* sp. nov.

Female (holotype, range for paratypes) (Figure 5). *Gnathosoma.* Chelicerae 95 (75–85) long; swollen proximal part of cheliceral base 45 (35–40) long, slender distal part 50 (40–50) long. Movable cheliceral digit three-pronged; fixed cheliceral digit spinous, approximately 5 (5) long. Palpal femur with serrate seta *dF* 25 (20–25) long; palpal genu with filiform smooth seta *dG* 30 (25–35) long. Palpal tibia with 3 smooth setae (*dTi*, *l’Ti*, *l”Ti*) and slender curved claw. Palpal tarsi with 3 smooth setae. Subcapitular seta *n* filiform, smooth, 25 (25–30) long. Each branch of peritremes with barely visible chambers, 55 (55) long. Hypostome with three-pronged apex. *Idiosoma* 275 (290–380) wide and 210 (240–340) long. Dorsum. Propodonotal shield well outlined, with minute punctuation in medial part, very slightly concave anteriorly and posteriorly, 95 (100–105) wide in anterior part, 70 (80–85) long in middle part; bearing six pairs of very slightly plumose, thick, blunt-pointed setae, 20–40 long; one seta situated medially shorter, about 10 (10–15) long. Posteriorly and laterally to propodonotal shield numerous setae (about 38 pairs), less serrate than those on shield, 20–40 long. Eyes present laterally to propodonotal shield on small plate with one serrate seta 40 long. Venter. Anterio-medial part with 4 rows (14 setae, 10–15 long) of plumose antero-median short setae (11–13 setae in paratypes); below, in posterior half of idiosoma, several rows of slightly serrate, thicker tapered setae (96 in holotype, 70–86 in paratypes), 40–55 long. *Genital region.* Genital setae represented by four pairs of slender, slightly serrate setae *g1–g4* situated dorsally; *g1*, *g2* about 20 (25) long, *g3–g4* about 10 long. Pseudanal series represented by 3 pairs of blunt-pointed, flattened, slightly serrate setae *ps1–ps3*, about 40, 35, and 25 long, respectively; *ps1* and *ps2* situated dorsally, *ps3* ventrally. *Legs.* Coxal setation: *1a*, *1b*, *2a*, *2b*, *3a*, *3b*, *3c*, *3d*, *4a*, *4b*, *4c* arranged in formula: 2–2–4–3. Setae *1a*, *1b* filiform, smooth; *2a*, *2b*, *3a*, *3b*, *4a*, *4b* thick, plumose. One short plumose seta present between coxal plates II and antero-median setae. Leg chaetotaxy of tibiae I–IV (5–5–5–5), genua I–IV (0–0–0–1), femora I–IV (3–2–2–2), trochanters I–IV (1–1–1–1). Setae *vTrI–IV* and *dFI* serrate; *ldFII–IV*, *vFII–IV*, *l’TII–IV*, *dTI–IV*, *vdTI–IV* long and smooth; *vFI* filiform with barely discernible serration. Setation of tarsi I: 14 setae (*ft*, *tc*’, *tc*”, *p*’, *p*”, *a*’, *a*”, *it*’, *it*”, *u*’, *u*”, *vs*’, *vs*”, *pl*’) and solenidion *w1*; tarsi II: 10 setae (*tc*’, *tc*”, *p*’, *p*”, *a*’, *a*”, *u*’, *u*”, *vs*’, *vs*”) and *w1*; tarsi III, IV: 10 setae each (*tc*’, *tc*”, *p*’, *p*”, *a*’, *a*”, *u*’, *u*”, *vs*’, *vs*”). Solenidion *w1* (about 25 long) longer than seta *ft* (about 5 long). Setae *tc*’, *tc*”, *it*’ and *it*” of leg I represented by eupathidia; *tc*’, *tc*” of legs II–IV, *u*’, *u*”, *vs*’, *vs*”, *a*’, *a*”, and *pl*’ of legs I–IV filiform.

Imagochrysalis. *Gnathosoma* barely discernible, inserted at ventral surface of idiosoma. Peritremes with barely discernible chambers. *Idiosoma* 300 long, 300 wide. Only coxae I and II visible.

Larva (Figure 4c). *Gnathosoma* as in female. Chelicerae about 55 long; slender cheliceral part 25 long, swollen basal part about 30 long. Setae *dF* serrate, 30 long; setae *dG* 40 long. Peritremes about 35 long. Subcapitular setae *n* absent. *Idiosoma* almost rounded, 150–265 long and 170–275 wide. Dorsum. Propodonotal shield 65–70 long and 50–55 wide; well-outlined, bearing four pairs of densely serrate setae: 2 pairs situated antero-laterally (1 pair longer, 45 long, 1 pair shorter, 20 long), and 2 pairs situated posteriorly on the shield, both about 45 long. Laterally to propodonotal shield eye on ocular plate present and accompanied by 1 seta. In posterior half of idiosoma five pairs of posterior setae 40–50 long present. *Venter* devoid of any setation. Genital area with 3 filiform genital setae *g1–g3*, about 5–10 long, and 2 slightly serrate pseudanal setae *ps1–ps2* 15–20 and 20–25 long. Coxae in formula: 2–0–1. Setae *1a*, *1b* filiform; *3a* short, densely serrate. Setation of trochanters–tarsi I–IV as in female, except for presence of *dGI* in several specimens; setation of legs I–III typical for pterygosomatidlarva (Figure 5 and Table 2 in [21]).

Differential diagnosis. *G parva* n. sp. is most similar to *Geckobia bochkovi* Fajfer, 2023 from *Ptyodactylus guttatus* (Heyden) from Israel [22]. In females of both species, the propodonotal shield is well outlined and slightly concave in anterior and posterior part of idiosoma, palp seta *dG* and subcapitular setae *n* are filiform and smooth, eyes are present laterally to propodonotal shield, in the medial and posterior part of the idiosomal venter lanceolate setae are present, coxal setae *1a* and *1b* are filiform, whereas setae *2b* and *3c* are thick and densely serrate, and four pairs of genital setae are present. In this new species, the idiosoma is much smaller (275–380 wide and 210–340 long) and propodonotal shiels bears six pairs of setae, leg seta *lGI* is absent, coxal setae *3d* is present, pseudanal series is represented by three pairs of setae *ps*. In *G. bochkovi* the idiosoma is much bigger (560–650 wide and 520–650 long), the propodonotal shield bears 14 pairs of setae, leg seta *lGI* is present and coxal seta *3d* is absent, the pseudanal series is represented by 12 pairs of setae *ps*.

Type material. Female holotype, 1 imagochrysalis and 9 larvae from *Ptyodactylus puiseuxi* Boutan, (HUJ no. 18259) (tympanum), Jordan: Wadi Khalid, 16.01.1987, coll. Yaacov Pesach; 1 female from same host (HUJ no. 18258) (tympanum), Israel, Northern district, Golan: Qazbiya, 24.05.1987, coll. Yehudah L. Werner; 3 females, 3 imagochrysalis, and 16 larvae from same host (HUJ no. 18672), Israel, Northern District, Golan: foot of Rekhes Bashanit, 24.05.1987, coll. Yehudah L. Werner.

Type material deposition. Holotype female, 2 imagochrysalis, 10 larvae in the HUJ (HUJINV-Acari_Pte00004.1–13), 3 female paratypes, 1 imagochrysalis, and 14 larvae in the CSWU (CSWU-Pte0020.1–18).

### 3.2. Ecological Analyses

A total of 1135 *Ptyodactylus* specimens were examined from museum collections. Among these, 37 hosts (3.26%, 95% CI: 2.31–4.47%) were infected with five *Geckobia* species with species-specific prevalence patterns (host-level, 95% CI) as shown in Table 1. A total of 264 individual mites were collected. Intensity averaged 7.14 ± 8.46 with a median of 4.0 (IQR 1.0–10.0), while abundance was 0.233.

Co-infections with multiple *Geckobia* species occurred in 5 of 37 infected hosts (13.5%). The most frequent combinations were *G. bochkovi* + *G. synthesys* (three hosts), *G. bochkovi* + *G. squameum* (one host), and *G. inermis* + *G. squameum* (one host). Single-species infections predominated (32 hosts, 86.5%).

Host species analysis across all 1135 examined specimens revealed differential infection patterns (see host species-specific prevalence in Table 2). *Ptyodactylus guttatus*, the dominant species (904 hosts, 79.6%), showed 27 infections, 2.99% prevalence (95% CI: 1.88–4.10%), with a mean intensity of 5.44 ± 6.41 mites per infected host. *Ptyodactylus puiseuxi* (177 hosts) exhibited seven infections, 3.95% prevalence (95% CI: 1.08–6.83%), with a higher mean intensity of 14.29 ± 12.59. *Ptyodactylus hasselquistii* showed two infections out of 41 hosts (4.88% prevalence, 95% CI: 0.00–11.47%) and a mean intensity of 1.50 ± 0.71. Unidentified species *Ptyodactylus* sp. exhibited zero infections (0/12 hosts, 0.00% prevalence [95% CI: 0.00–0.00%]). Among infected hosts, mean intensity differed markedly between *P. puiseuxi* and *P. guttatus* (Hedges’ g = 1.12, 95% CI 0.24–1.99; large effect).

Exact binomial tests revealed significant deviations from 1:1 sex ratios in three species (*G. squameum*, *G. synthesys*, and *G. parva*) before multiple comparison correction. After Holm correction for multiple comparisons, only *G. squameum* showed significant female bias (31♀:9♂, *p* = 0.0034, Cohen’s h = 0.73 [large effect], 95% CI: 0.31–1.15, power = 0.94) whereas *G. synthesys* (16♀:5♂, raw *p* = 0.0266, corrected *p* = 0.1064) and *G. parva* (6♀:0♂, raw *p* = 0.0312, corrected *p* = 0.0938) showed trends toward female bias but were not significant after correction (Table 3). *G. bochkovi* (21♀:16♂, *p* = 1.0000) and *G. inermis* (2♀:2♂, *p* = 1.0000) showed balanced sex ratios.

Environmental filtering showed a systematic prevalence decline: Mediterranean 4.3% (27/628 hosts), semi-arid 2.8% (8/288), desert-edge 1.1% (1/94). Due to low expected values in the desert-edge category, Fisher’s exact test confirmed a significant association between climate zone and infection status (*p* = 0.029). The climate effect (Cramér’s V = 0.078, 95% CI: 0.045–0.112) exceeded the host species effect (Cramér’s V = 0.015, 95% CI: 0.001–0.034) by 5.2-fold (95% CI: 2.3–11.7), confirming climate as the primary structuring force (χ^2^ = 8.74, df = 3, *p* = 0.033, 1-β = 0.60). The systematic prevalence decline across the climate gradient supports the role of climate in shaping mite distributions (Cramér’s V = 0.078 vs. 0.015 for host species). Pairwise risk ratios (RRs) for prevalence across climate zones were as follows: Mediterranean vs. desert-edge RR = 4.04 (95% CI 0.56–29.4), Mediterranean vs. semi-arid RR = 1.55 (0.71–3.36), and semi-arid vs. desert-edge RR = 2.61 (0.33–20.6); overall association strength, Cramér’s V = 0.078 (95% CI 0.045–0.112).

#### Seasonal Phenological Plasticity

**Figure 6:**
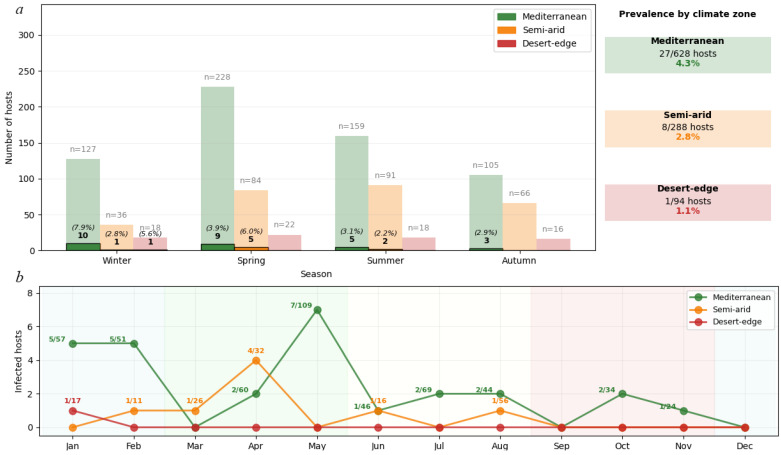
(**a**) Seasonal infection patterns showing systematic prevalence decline from Mediterranean (4.3%) through semi-arid (2.8%) to desert-edge zones (1.1%). Light bars show hosts examined per season; dark bars show infected hosts with prevalence percentages. Sample sizes (n) represent hosts examined per season. (**b**) Monthly infection dynamics revealing phenological plasticity: Mediterranean populations peak in winter (January–February), while semi-arid populations show spring-shifted activity (April–May), reflecting adaptation to local climate constraints. Numbers above the points indicate infected/examined host ratios. Desert-edge populations show minimal sporadic activity. Climate zones classified using the Köppen–Geiger system with 95% concordance to published Israeli climate boundaries. Analysis based on 1010 hosts with complete climatic data.

*Geckobia* mites exhibited distinct seasonal activity patterns (Figure 6) that varied systematically across climate zones. Mediterranean populations showed pronounced winter activity peaks, with the highest infection rates in winter (10/127 hosts, 7.9%), followed by spring (9/228 hosts, 3.9%), summer (5/159 hosts, 3.1%), and autumn (3/105 hosts, 2.9%). Peak reproductive activity occurred during the mild, humid Mediterranean winter when temperature–moisture conditions optimize mite survival and inter-host transmission.

In contrast, semi-arid populations demonstrated spring-shifted phenology, with maximum activity in spring (5/84 hosts, 6.0%) compared to winter (1/36 hosts, 2.8%) and summer (2/91 hosts, 2.2%). No infections were detected in semi-arid autumn samples (0/66 hosts, 0.0%). This temporal shift reflects adaptation to the brief post-winter period before extreme summer desiccation, when conditions briefly achieve suitable humidity levels for the mite’s survival and reproduction.

Developmental stage composition varied seasonally across climate zones. Summer months (June–August) showed exclusively adult-only infections with no eggs or juvenile stages detected, despite examining 268 hosts during this period. In contrast, winter (Mediterranean) and spring (semi-arid) infection peaks coincided with maximum juvenile presence (larvae and nymphs comprising 35–53% of populations in Table 3), indicating active reproduction.

Desert-edge populations showed minimal and sporadic activity, with infections detected only in winter (1/18 hosts, 5.6%), while spring (0/22 hosts), summer (0/18 hosts), and autumn (0/16 hosts) showed complete absence despite adequate sampling effort. This pattern indicates that climatic conditions rarely reach thresholds suitable for sustained mite reproduction in hyperarid environments.

**Figure 7:**
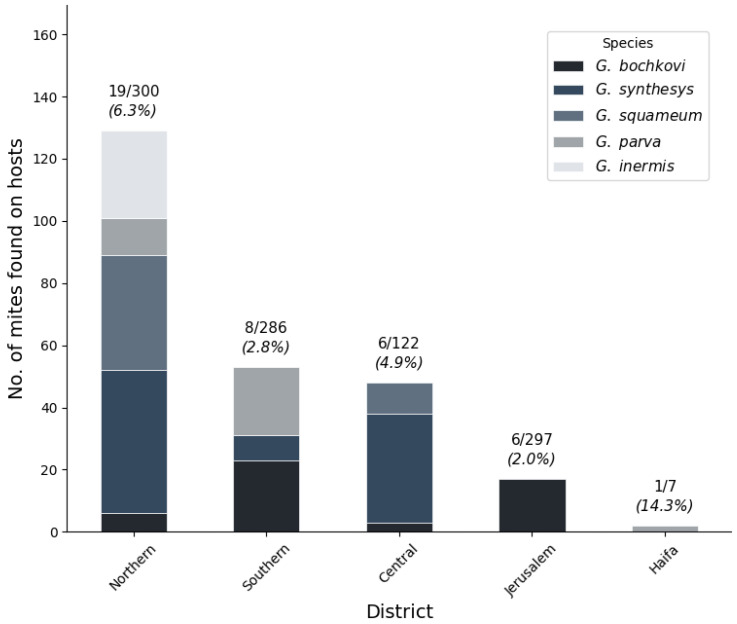
Geographic distribution of *Geckobia* species across Israel and West Bank. Mite abundance by district showing total number of individuals found per district, with infected–total host ratios and prevalence percentages above bars.

Analysis of mite distribution across Israeli administrative districts (Figure 7) revealed significant geographic heterogeneity in both prevalence and species composition. Among hosts with district assignments, infections were recorded from all five main administrative districts, with the highest prevalence in Northern District (19/300 hosts, 6.3%) followed by Central District (6/122 hosts, 4.9%), Southern District (8/286 hosts, 2.8%), Jerusalem District (6/297 hosts, 2.0%), and Haifa District (1/7 hosts, 14.3%). The small sample size in Haifa District (n = 7) limits interpretation.

*Geckobia* species showed distinct district-level distribution patterns. *G. bochkovi* demonstrated the broadest geographic range, occurring in four districts, with the highest abundance in Southern District (23 individuals from five hosts, mean intensity = 4.6) (Figure 7). *G. synthesys* showed preference for northern and central regions, with the highest concentrations in Central District (35 individuals from four hosts, mean intensity = 8.8) and Northern District (46 individuals from five hosts, mean intensity = 9.2). *G. squameum* was restricted primarily to Northern District (37 individuals from seven hosts, mean intensity = 5.3) with limited occurrence in Central District. *G. parva* occurred mainly in Northern and Southern Districts, while *G. inermis* was recorded only from Northern District (28 individuals from one host).

Mite abundance (total individuals per host examined) varied considerably across districts: Northern District (0.430), Central District (0.393), Haifa District (0.286), Southern District (0.185), and Jerusalem District (0.057). The 7.5-fold difference in abundance between Northern District (predominantly Mediterranean climate: >600 mm rainfall, 10–20 °C) and Jerusalem District (semi-arid transition: 200–400 mm rainfall, warmer temperatures) suggests climate-driven constraints on mite establishment, consistent with our environmental filtering analysis.

**Figure 8:**
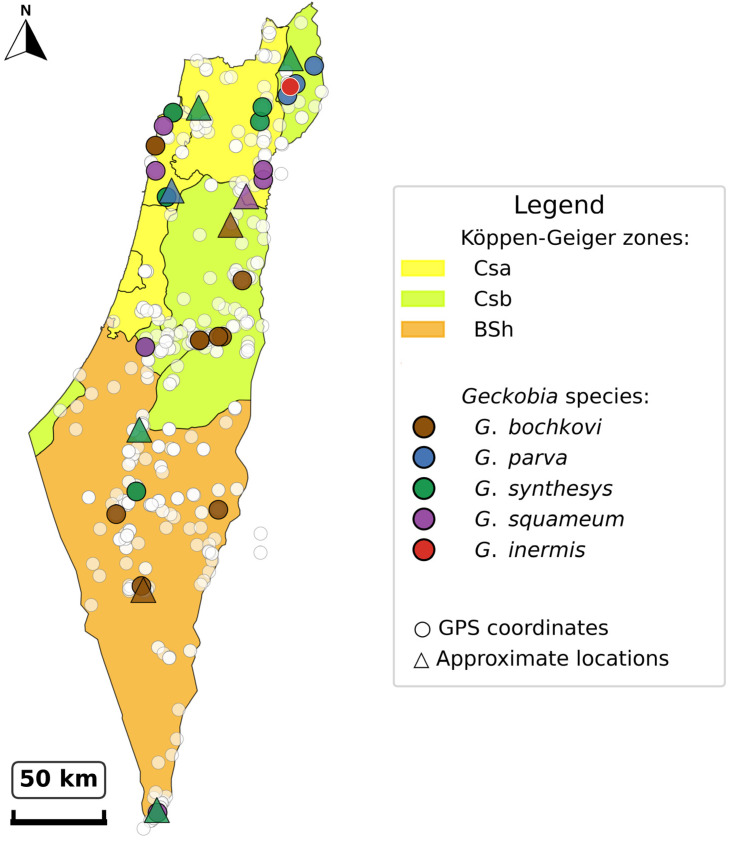
Distribution map showing all infected host locations. Map achieves >95% coverage of infected hosts from museum collections (1965–1991), revealing species-specific distribution patterns and concentration in northern Mediterranean climate zones (Csa, Csb), followed by occurrences in semi-arid (BSh) and desert-edge (BWh) areas. Jitter applied to overlapping coordinates to prevent visual obstruction while maintaining spatial accuracy within a 0.03° radius.

Co-infections with multiple *Geckobia* species were most frequent in Northern District, where 5 of 16 infected hosts (31.3%) harbored more than one species (Figure 8). Analysis of realized versus potential geographic ranges revealed incomplete exploitation of host distributions. While Mediterranean and semi-arid zones showed consistent mite occurrence across the sampled range, desert-edge zones exhibited patchy and sporadic infections despite substantial host availability. This pattern indicates that climate sets hard boundaries on mite distribution, creating “climate refugia” where mites persist only under locally favorable conditions.

## 4. Discussion

Our Israeli *Geckobia* fauna represents the southeastern terminus of the Eastern Mediterranean pterygosomatid assemblage documented by Bertrand et al. [16] and Eren and Açıcı [18]. Bertrand et al. ([16], Figure 4) reviewed *Geckobia* mites with scale-like ventral setae across the entire Mediterranean Basin, identifying distinct western (*G. estherae*; *G. loricate* Berlese, 1892; and *G. latasti* Mégnin, 1878 on *Tarentola* from Malta, Iberia, North Africa, and the Canary Islands) and eastern assemblages (our Israeli species plus *G. sharygini* from Crimea and *G. turkestana* from Turkey; Supplementary Table S2), separated by biogeographic and host-family boundaries. The regional distribution pattern suggests host-family-structured parasite assemblages rather than strict geographic segregation: species associated with Phyllodactylidae (*Ptyodactylus* in the southern Levant; *Tarentola* in the western Mediterranean: *G. loricata*, *G. latastei*, and *G. estherae* [16]) versus species associated primarily with Gekkonidae. For example, Eren and Açıcı ([18], Table 3) reported *G. turkestana* from *Mediodactylus* cf. *kotschyi* in northeastern Turkey (Artvin Province). The distributional gap between our Israeli assemblage and documented Turkish *Geckobia* [18] likely reflects host-family boundaries rather than dispersal limitations, as Gekkonidae-associated species (*G. turkestana*) and Phyllodactylidae-associated species (our taxa) show non-overlapping distributions corresponding to their respective host ranges. Additionally, Jabbarpour [38], cited in Eren and Açıcı ([18], Table 3), documented broader Turkish records for *G. turkestana* on multiple gekkonid genera (*Tenuidactylus russowi*, *Cyrtopodion scabrum*, *Hemidactylus turcicus*, and *Mediodactylus* cf. *kotschyi.)* with occasional records from lacertids (*Apathya cappadocica*; *Darevskia dryada*) and for *G. tarentulae* on *H. turcicus* and *A. cappadocica*. This gekkonid-associated fauna extends the distribution documented by Bertrand et al. [17] for Crimean–Central Asian populations and is morphologically distinct from our *Ptyodactylus*-parasitizing species (see Supplementary Table S2).

The overall prevalence of *Geckobia* spp. on *Ptyodactylus* spp. (3.26%) was substantially lower than reported for tropical gecko populations. Budianto and Basuki [15] documented 28% overall prevalence in Indonesian *Hemidactylus* spp., with species-specific values reaching 100% for *G. bataviensis* Vitzhum, 1926 (=*G. gleadovania*) on *H. frenatus* Dumeril and Bibron, 1836 and *G. diversipilis* Hirst, 1926 on *H. platyurus* (Schneider, 1797). Similarly, research by Díaz et al. [25] recorded 80% infestation rates in Colombian *H. frenatus*, with *G. keegani* Lawrence, 1953 (76.6%) and *G. bataviensis* (50%) showing mean intensities of 11.6 and 9.66 mites per host, respectively. Conversely, broader Indonesian studies by Prawasti et al. [26] (25 sites, 448 geckos) reported 221/448 infested hosts (~49%) and highlighted how host skin morphology (e.g., fewer skin folds in *H. platyurus*) can decrease prevalence locally.

**Figure 9:**
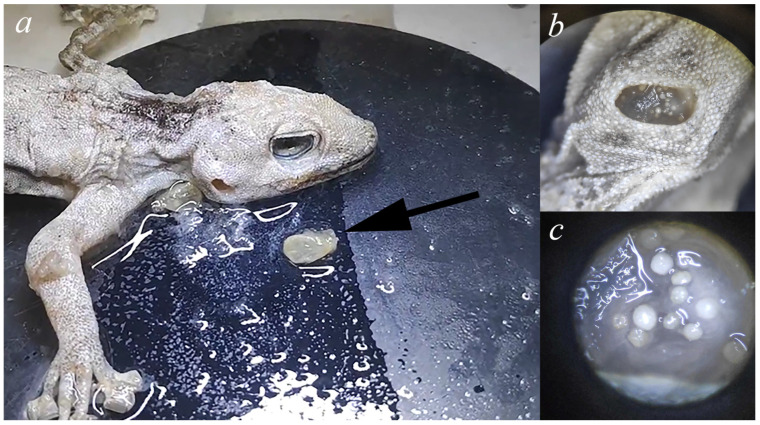
*Geckobia* mites found on *Ptyodactylus guttatus* species. (**a**) *Ptyodactylus guttatus* with removed double skin plug (arrow), which blocked tympanic cavity entrance, creating humid microenvironments favorable for mite survival. (**b**) Tympanic cavity of infected host after removal of retained skin. (**c**) *Geckobia* mites visible within the tympanic cavity (magnified view), individual mites around 200–400 μm in length.

This dramatic difference in prevalence between tropical (28–100%) and arid systems (3.26%) reflects fundamental limitations imposed by environmental stress on parasite survival. Arid ecosystems are characterized by extreme temperature fluctuations, low humidity, and unpredictable precipitation patterns that create physiological challenges for arthropod parasites. To overcome desiccation stress, *Geckobia* mites in our study exhibited pronounced microhabitat specialization. The vast majority of mites (94.6%, 250 of 264 individuals) were found in the tympanic cavity, which protects against both desiccation and mechanical removal by host grooming. Only 5.4% (14 individuals) occurred in other body locations: beneath dorsal scales (n = 8, 3.0%), axillae (n = 4, 1.5%), and between digits (n = 2, 0.8%). Notably, we found, for the first time, a “double skin plug” at the ear entrance—two layers of retained ecdysis closing the tympanic opening (Figure 9), with mites present both between the skin layers and inside the tympanum. Because this skin shed was observed in the majority of infected hosts (35/37 hosts) and it was absent in all checked uninfected hosts (0/1098 examined), it suggests a direct association between mite presence and altered ecdysis patterns in the ear region. The mechanism causing skin retention remains unclear—it may result from mechanical impediment by mite aggregations preventing normal sloughing. We hypothesize that the retained skin creates a sealed microenvironment that likely creates more stable microclimatic conditions compared to exposed dorsal surfaces on the host’s body. Other studies on pterygosomatids have shown that the mite species did not come into direct competition and were associated with different body regions while associated with the same host species [7,39]. Consistent with this, in all co-infections where body site was known (4/4; “jar” record excluded), both species (*G. bochkovi* + *G. synthesys*) occurred in the tympanum. Unlike tropical geckos, where mites use many body regions of hosts to avoid direct competition, in Israeli *Ptyodactylus*, the ear is basically the only safe place that keeps juveniles from drying out, which may explain the rarity of co-occurrence and the low mite loads. Accordingly, our material juvenile-stage peaks differed between the two species—*G. synthesys* showed winter–spring recruitment, whereas *G. bochkovi* peaked in autumn—indicating asynchronous reproduction and reducing temporal niche overlap. The low co-infection rate (13.5%) likely reflects the combination of limited ear cavity space and low overall mite density in arid environments, though our sample size (n = 5 co-infections) precludes statistical testing of competition hypotheses.

Within desert-edge zones (BWh classification, n = 94 hosts examined), the single infected host originated from the northern Negev transition zone (31.2° N) near the semi-arid boundary, not from hyperarid core desert regions (29–30° N), where zero infections were found despite examining 18 hosts. This indicates that mites do not penetrate true hyper-arid environments, which indicates that abiotic factors, such as thermal and hydric stress, outweigh host availability in determining parasite occurrence. *Geckobia* mites complete their entire life cycle on the host surface, with all developmental stages remaining permanently attached to their gecko hosts. However, inter-host transmission likely occurs during direct host contact, particularly during mating encounters, territorial interactions, or shared refugia use, when geckos aggregate in favorable microhabitats. Notably, *Ptyodactylus* geckos are highly effective behavioral thermoregulators that seek rock crevices and shaded refugia during temperature extremes. This behavior creates predictable thermal environments for associated parasites, potentially explaining how mite populations persist in desert-edge zones despite apparent habitat unsuitability.

Another remarkable example of phenological plasticity of *Geckobia* mites in response to local climate regimes is a documented 3–4-month shift in peak reproductive timing between Mediterranean (winter) and semi-arid (spring) populations. This suggests that *Geckobia* populations possess developmental flexibility to exploit narrow environmental windows across diverse climate zones. The Mediterranean winter peak (January–February) coincides with optimal temperature–humidity combinations (10–15 °C, 60–80% humidity) that facilitate mite survival and inter-host transmission. Conversely, the semi-arid spring peak (April–May) exploits the brief post-winter period before extreme summer desiccation, when soil moisture from winter rains creates temporarily suitable microclimatic conditions. In summer, prevalence is low, eggs are not detected, and infections are predominantly adult-only across climate zones; despite abundant host availability, this pattern points to physiological (water balance) constraints rather than a simple reduction in host activity. This contrasts sharply with tropical *Geckobia* populations that maintain year-round activity under stable, humid conditions.

Geographic analysis reveals incomplete exploitation of host ranges by *Geckobia* species, providing further evidence for climate-driven filtering. While *P. guttatus* occurs throughout the Negev Desert southward to the Sinai Peninsula [19], *G. bochkovi* infections were restricted to northern portions of this range (northernmost Negev, Figure 8), demonstrating that absolute climatic thresholds limit mite distributions even where hosts persist. Similarly, although *P. puiseuxi* ranges across the Mediterranean Levant (Lebanon, Syria, and northern Israel) [20], *G. parva* and *G. inermis* were found only within Israeli populations. The mite-free southern populations of *P. guttatus* (hyper-arid core, Negev, 29–30° N), despite host availability, confirm that climate, not host presence, determines mite occurrence at range margins. In contrast, *G. synthesys* exhibits broader climate distribution (Mediterranean through desert-edge zones), indicating either superior physiological tolerance to desiccation stress or greater behavioral flexibility in microhabitat selection.

The observed female bias in *G. squameum* (77.5% females, *p* = 0.0034 after Holm correction) represents the first documented sex ratio skew in this species and is consistent with patterns reported in other Pterygosomatidae [7]. Female-biased populations may result from several non-exclusive mechanisms. Firstly, males have never been observed in at least half of all known pterygosomatid species, suggesting that they may reproduce through parthenogenesis [4]. The absence of males in *G. parva* populations, while not statistically significant after correction (*p* = 0.0938), may indicate facultative parthenogenesis [13]. Secondly, we can suspect that the sex ratio biases observed in the scale mites are induced by *Wolbachia*, endosymbiotic bacteria that manipulate arthropod reproduction by killing males, feminizing genetic males, or inducing parthenogenesis to enhance their maternal transmission [7,40].

Although our study represents the first comprehensive analysis of *Geckobia* ecology in Mediterranean–desert systems, several limitations should be acknowledged. Firstly, the temporal scope of museum collections (1965–1991) may not capture recent climate-driven changes in distribution or phenology. Secondly, curation can also affect detection: mites on exposed skin surfaces are more likely to be lost during museum preparation; however, our focus on tympanic cavity inhabitants (94.6% of detections) minimizes this bias, as these mites remain firmly attached to protected microhabitats. The consistency of our key patterns—ear microhabitat use, rare co-infections, and seasonality—is unlikely to be a result of preservation artifacts.

Thirdly, while our focus on environmental filtering provides insights into abiotic constraints, we did not systematically analyze host phylogenetic relationships, which may contribute additional explanatory power for understanding species-specific distribution patterns. This matters because phylogenetic work on other scale mites, i.e., *Pterygosoma* [23], shows high host and topical specificity and host–parasite cophylogeny; thus, developing a comparable phylogenetic framework for *Geckobia* would likely add explanatory power to species-level patterns. Moreover, *Geckobia* species used as outgroups in phylogenetic studies of the above-mentioned *Pterygosoma* mites were rendered paraphyletic in the analysis, highlighting the need for phylogenetic studies of this genus. Additionally, the recent phylogenetic revision of *Ptyodactylus* [20] has significant implications for interpreting our host–parasite associations. The resurrection of *P. bischoffsheimi* and clarification of *P. puiseuxi* sensu stricto distributions indicate that several historical “*P. guttatus*” records from northern Israel likely represent misidentified *P. puiseuxi.* Specifically, for *G. bochkovi*, while the holotype from Be’er Sheva (HUJ 18802) correctly represents *P. guttatus*, northern paratypes (HUJ 7225, 2798, and 11033), and non-type material (HUJ 2915 and 2916) originate from localities now recognized as within the range of *P. puiseuxi s.s.*, not *P. guttatus*. These specimens require molecular verification to confirm host identity.

Importantly, this taxonomic uncertainty affects species-level distribution details but does not undermine our main finding of climate-driven environmental filtering. Our central conclusion—that climate effects exceed host species effects in structuring mite distributions—remains robust regardless of host taxonomy, as it compares broad climate zones rather than individual host species. For example, the apparent “broad distribution” of *G. bochkovi* across climate zones may actually represent two distinct host–parasite associations: *G. bochkovi* on *P. guttatus* in southern arid zones and potentially a cryptic lineage on *P. puiseuxi* in northern Mediterranean zones. Given that pterygosomatids show high host specificity [23], parallel cryptic diversity in both hosts and parasites is plausible. The 37 infected hosts in our dataset may, therefore, represent more complex host–parasite relationships than initially apparent. Future studies should prioritize molecular barcoding of both preserved host specimens and their associated mites to resolve these taxonomic ambiguities and test for cryptic parasite lineages associated with the newly recognized *Ptyodactylus* species.

Finally, the moderate statistical power (60%) for detecting climate effects reflects the ecological reality of studying rare parasitic infections in arid environments. This pattern aligns with broader ecological principles, where parasite diversity and abundance decrease systematically along aridity gradients, as demonstrated by our spatial (Figure 6) and temporal (Figure 8) distribution analyses. Álvarez-Ruiz et al. [41] found that ectoparasite loads in *Psammodromus algirus* (Linnaeus, 1758) decreased along elevational gradients toward harsher conditions, while [42] showed that environmental variation mediates mite and tick prevalence in *Zootoca vivipara* (Lichtenstein, 1823), with microclimate and habitat structure being key drivers. Similarly, Drechsler et al. [43] demonstrated that phenological patterns of mite infections in Mediterranean lizard communities track local environmental conditions. The observed 3.9-fold prevalence decline from Mediterranean (4.3%) to desert-edge zones (1.1%) falls within expected ranges for reptilian ectoparasites in xeric environments, where low humidity limits on-host survival of eggs and immatures and narrows opportunities for host-to-host transfer. While our sample of 1135 hosts exceeds most published *Geckobia* studies, achieving 80% power for small effects would require approximately 1680 hosts with proportional sampling across climate zones—logistically challenging given inherently low desert infection rates. The observed effect size (Cramér’s V = 0.078, 95% CI: 0.045–0.112), though statistically “small,” represents a biologically meaningful pattern consistent with fundamental water balance constraints on arthropod physiology in arid environments.

## 5. Conclusions

This study provides the first quantitative analysis of pterygosomatid mite distributions across an aridity gradient, revealing how environmental constraints shape parasite communities in xeric ecosystems. We documented a 3.9-fold decline in *Geckobia* prevalence from Mediterranean (4.3%) to desert-edge zones (1.1%) in Israel, demonstrating that climate acts as the primary filter for these ectoparasites.

Our studies show that three key adaptations enable *Geckobia* persistence in water-limited environments: concentration in tympanic cavities (94.6% of mites) and formation of a “double skin plug” that seals ear openings, maintaining humidity critical for mite survival, and phenological plasticity, with populations shifting peak activity from winter in Mediterranean zones to spring in semi-arid regions. The rarity of co-infections (13.5%) and asynchronous juvenile development among species suggests intense competition for limited favorable microhabitats on the host’s body.

These conclusions are most robust for broad-scale patterns (climate zone effects, overall microhabitat preferences, seasonal phenological shifts) supported by large sample sizes (1010 hosts across three zones, 264 mites). However, detailed conclusions about specific months, sites, or rare species must be interpreted cautiously, given low infection rates (3.26% overall) and sampling limitations, with some specific months and localities yielding only single infected hosts over the 26-year period.

These findings have implications for predicting parasite responses to climate change in arid regions. Comparative data from other arid systems are lacking, as most Pterygosomatidae studies focus on tropical or subtropical regions, making our results particularly valuable for understanding parasite ecology in water-limited environments.

## Figures and Tables

**Table 1:** Species-specific parasitological parameters for *Geckobia* spp. on *Ptyodactylus* spp.

Species	No. of Infested Hosts	Prevalence (%) [95% CI]	Abundance	Intensity (Mean ± SD, Median)	Cramér’s V [95% CI]	Power
*G. bochkovi*	16	1.41 [0.87–2.28]	0.043	3.06 ± 2.61 (2.0)	0.041 [0.018–0.064]	0.52
*G. synthesys*	12	1.06 [0.61–1.84]	0.084	7.92 ± 5.96 (6.0)	0.035 [0.012–0.058]	0.71
*G. squameum*	9	0.79 [0.42–1.50]	0.049	6.22 ± 5.24 (4.0)	0.029 [0.008–0.050]	0.48
*G. parva*	4	0.35 [0.14–0.90]	0.032	9.00 ± 8.46 (6.5)	n.d. */−	<0.20
*G. inermis*	1	0.09 [0.02–0.50]	0.025	28.0 ± 0.0 (28.0)	n.d. */−	n.a. *

* n.d. = not determined due to insufficient sample size (n < 5 hosts); n.a. = not applicable.

**Table 2:** Host-specific parasitological parameters for *Geckobia* spp. on *Ptyodactylus* spp. Abbreviations: No—number of infested hosts; T—total number of hosts checked for mites.

Species	No	T	Prevalence (%) [95% CI]	Abundance	Intensity (±SD)
*P. guttatus*	27	904	2.99 [1.98–4.32]	0.167	5.59 ± 6.63
*P. puiseuxi*	7	177	3.95 [1.60–7.98]	0.588	14.86 ± 12.64
*P. hasselquistii*	2	41	4.88 [0.60–16.53]	0.073	1.50 ± 0.71
*P. oudrii*	1	1	100.00 [2.50–100.00]	6.000	6.00 ± 0.00

**Table 3:** Species composition and developmental stage structure of *Geckobia* mites. *Abbreviations*: T—total number of mites; A—total adults; F—females; M—males; L—larvae; D—deutonymphs; P—protonymphs; C—chrysalids (imagochrysalis + nymphchrysalis combined), (M:F)—sex ratio (male–female). *p*-values from chi-square goodness-of-fit tests assuming 1:1 sex ratio. Bold *p*-values indicate significant deviation from a 1:1 ratio (*p* < 0.05).

Species	T	A	F	M	L	D	P	C	M:F	Raw *p*-Value	Holm *p*-Value
*G. bochkovi*	49	37	21	16	9	0	0	3	0.76	0.5114	1.0
*G. synthesys*	95	21	16	5	31	15	15	13	0.31	0.0266	0.1064
*G. squameum*	56	40	31	9	5	0	3	8	0.29	**0.0007**	**0.0034**
*G. parva*	36	6	6	0	25	1	0	4	0.0	0.0312	0.0938
*G. inermis*	28	4	2	2	12	5	7	0	1.0	1.0	1.0
*Total*	264	108	76	32	82	21	25	28	0.42	-	-

## Data Availability

The mite material stored in the Cardinal Wyszynski University in Warsaw (Warsaw, Poland) and all data supporting the study will be shared upon reasonable request to Monika Fajfer-Jakubek.

## Supplementary Materials

The following supporting information can be downloaded at: https://www.mdpi.com/article/10.3390/ani15233461/s1, Table S1: Summary of mite species, sex ratios, developmental stages and host/locality data used in the study. Table S2: Comparative summary of *Geckobia* species from the Eastern Mediterranean and adjacent regions (Turkey, Levant, Egypt, Crimea), representing the most geographically and ecologically relevant congeners of the newly described Israeli taxa. Based on Bertrand et al. [1] Figure 4, which reviewed scale-like seta-bearing *Geckobia* across the entire Mediterranean Basin. Western Mediterranean species (*G. estherae* from Malta, *G. loricata* and *G. latastei* from Iberia/North Africa, *G. canariensis* and *G. tinerfensis* from Canary Islands) parasitizing *Tarentola* hosts are discussed in the main text but excluded from detailed morphological comparison as they represent a distinct biogeographic assemblage separated by >2000 km from our study area. *Legend of main climate zones (Köppen–Geiger classification)*: Csa—hot-summer Mediterranean climate, Csb—warm-summer Mediterranean climate, BWh—hot desert climate, BWk—cold desert climate, BSh—hot semi-arid climate (hot steppe), BSk—cold semi-arid climate (cold steppe), Cfa—humid subtropical climate, Cfb—oceanic climate (temperate oceanic), Dsa—hot-summer continental climate with dry summer, Dsb—warm-summer continental climate with dry summer, Dfa—hot-summer humid continental climate, Dfb—warm-summer humid continental climate. References [16,17,21,38,44,45] are cited in Supplementary Materials.
