# Supplementary material for: Population Structure and Climate Effects on Geckobia Infestation in Ptyodactylus Geckos from Israel and West Bank, with Descriptions of G. parva sp. nov. and G. inermis sp. nov
Source: Animals (Basel). 2025 Nov 30;15(23):3461. doi: 10.3390/ani15233461 (PMC12691030; doi:10.3390/ani15233461)
Supplement: Supplementary file 1 [file animals-15-03461-s001.zip › Supplementary _Table_2_.pdf]

Supplementary Table S2. Comparative summary of *Geckobia* species from the Eastern Mediterranean and adjacent regions (Turkey, Levant, Egypt, Crimea), representing the most geographically and ecologically relevant congeners of the newly described Israeli taxa. Based on Bertrand et al. [1] Figure 4, which reviewed scale-like seta-bearing *Geckobia* across the entire Mediterranean Basin. **Western Mediterranean species** (*G. estherae* from Malta, *G. loricata* and *G. latastei* from Iberia/North Africa, *G. canariensis* and *G. tinerfensis* from Canary Islands) parasitizing *Tarentola* hosts are discussed in the main text but excluded from detailed morphological comparison as they represent a distinct biogeographic assemblage separated by >2000 km from our study area. *Legend of main climate zones (Köppen–Geiger classification):* Csa – hot-summer Mediterranean climate, Csb – warm-summer Mediterranean climate, BWh – hot desert climate, BWk – cold desert climate, BSh – hot semi-arid climate (hot steppe), BSk – cold semi-arid climate (cold steppe), Cfa – humid subtropical climate, Cfb – oceanic climate (temperate oceanic), Dsa – hot-summer continental climate with dry summer, Dsb – warm-summer continental climate with dry summer, Dfa – hot-summer humid continental climate, Dfb – warm-summer humid continental climate.

***Geckobia* species**	**Host**	**Host Family**	**Locality / Country**	**Climate zone (Köppen–Geiger)**	**Group (Jack 1964)**	**Distinctive characters**	**Reference**
Geckobia tarentulae (Trägårdh, 1905)	*Tarentola annularis* (Geoffroy Saint-Hilaire, 1827)	Phyllodactylidae	Egypt	BWh, Csa, Csb BSk, Dsa	*latasti*	Gnathosoma: palpal setae *dF* serrate, *dG* filiform and smooth. Idiosoma slightly wider than long. Propodonotal shield barely outlined with 15 pairs of brush-like setae. Small eyes present. Setae located just behind shield brush-like. The remaining body setae are longer, with slightly serrated edges. Venter with anterior short brush-like setae; medial fan-shaped setae in posterior part become thinner and longer with slightly serrated margins. Genital area with 3 genital setae *g1–g3* and 3 pairs of pseudanal setae *ps1–ps3*.	[2, 3]
	Hemidactylus turcicus (Linnaeus, 1758)	Gekkonidae	Turkey				
	Apathya cappadocica (Werner, 1902)	Lacertidae	Turkey				
G. turkestana Hirst, 1926	*Tenuidactylus russowi* (Strauch, 1887)	Gekkonidae	former "Turkestan"*	BSk, BWk, Dfa, Dfb, Csa, BSh, BSk, Dsa, Cfb,	*latasti*	Gnathosoma: palpal setae *dF* short and densely serrate, setae *dG* slightly serrate. Idiosoma much wider than long. Propodonotal shield deeply concave in the posterior margin and with 6 pairs of brush-like setae. Small eyes present laterally on propodonotal shield. Dorsal setae slender and plumose. Venter. Anterior setae short and brush-like;, medial setae scale-like (heart-shaped), posterior setae oval or fan-shaped. Coxal setae in formula: 2–2–2–3. Genital area with 3 genital setae *g1–g3* and 8–9 pairs of pseudanal setae *ps1–ps9*.	[3, 4, 5]
	Mediodactylus cf. kotschyi (Steindachner, 1870)	Gekkonidae	Turkey				
	Hemidactylus turcicus (Linnaeus, 1758)	Gekkonidae	Turkey				
	Cyrtopodion scabrum (Heyden, 1827)	Gekkonidae	Turkey				
	Apathya cappadocica (Werner, 1902)	Lacertidae	Turkey				
	Darevskia dryada (Darevsky and Tuniyev, 1997)	Lacertidae	Turkey				
	Apathya cappadocica (Werner, 1902)	Lacertidae	Turkey				

G. squameum Bertrand, Paperna and Finkelman, 1999	*Ptyodactylus guttatus,* Heyden, 1827<br>*P. puiseuxi,* Boutan, 1893<br>*P. hasselquistii* Donndorff, 1798	Phyllodactylidae	Israel	BWh, Csa, Csb	*diversipilis*	Gnathosoma: palpal setae *dF* serrate, setae *dG* filiform and smooth. Idiosoma rounded. Propodonotal shield retrangular and weakly sclerotized with 8–9 pairs of setae. Small eyes with accompanied 1 setae present laterally to shield on ocular plate. Ventral setae modified into enlarged, imbricated scales. Coxal setae in formula: 2–2–2–3.	[6], this study
G. parvulum Bertrand, Paperna and Finkelman, 1999	Mediodactylus kotschyi (Steindachner, 1870)<br>*Cyrtopodion scabrum* (Heyden, 1827)<br>*Asaccus* spp.	Gekkonidae<br>Phyllodactylidae	Israel–Syria border<br>unknown	Csb, Dsb	**	Idiosoma roughly rhomboid. Propodonotal shield broad, quadrangular in shape, bordered anteriorly by two smooth plates with 3 pairs of setae; eyes small and inconspicuous. Venter in central part with transparent, foliaceous scale-like setae that transition from foliaceous scales to elongate setae posteriorly. Additional leg seta on trochanter III present.	[6], this study
G. sherygini Bertrand, Kukushkin and Pogrebnyak, 2013	*Mediodactylus danilewskii* (Strauch, 1887)	Phyllodactylidae	Ukraine (Crimeria)	Cfa, Cfb	*latasti*	Gnathosoma: palpal setae *dF* serrate, setae *dG* slightly serrate. Idiosoma triangular in shape, wider than long. Propodonotal shield semicircular, longer than wide with 7 pairs of setae. Small eyes present. Dorsum with plumose setae that increase in size from anterior to posterior part of idiosoma. Venter with 3-4 rows of short and stout anterior setae, medial region with scale-like setae gradually increasing in size posteriorly. Genital area with 3 genital setae *g1–g3*.	[7]
G. bochkovi Fajfer, 2023	*Ptyodactylus guttatus* Heyden, 1827	Phyllodactylidae	Israel	BWh, Csa	*latasti*	Gnathosoma: palpal setae *dF* filiform and with barely visible serration, setae *dG* filiform and smooth. Idiosoma rounded. Propodonotal shield lightly concave in its anterior and posterior part, with 14 pairs of slightly serrate setae. Small eyes present. Ventral surface bearing numerous anterior slightly serrate setae, gradually larger posteriorly; medial lanceolate setae wider medially than posteriorly. Coxal setae in formula: 2–2–3–3. Genital area with 4 pairs of setae *g1–g4* and 12 pairs of pseudanal setae *ps1–ps12.*	[8], this study
G. synthesys Fajfer, 2023	*Ptyodactylus guttatus* Heyden, 1827	Phyllodactylidae	Israel, Jordan, Egypt	Csa, Csb, BSh, BWh	*latasti*	Gnathosoma palpal setae *dF* with barely visible serration, setae *dG* filiform and smooth. Idiosoma rounded. Propodonotal shield squarish, with 6 pairs of slightly serrate setae. Small eyes present. Venter only with 19–28 pairs of filiform setae situated medially. Coxal setae in formula: 2–2–2–2. Genital area with 3 genital setae *g1–g3* and 2 pairs of pseudanal setae *ps1–ps2.*	[8], this study

G. parva sp. nov.	*Ptyodactylus puiseuxi* Boutan, 1893	Phyllodactylidae	Jordan, Israel	BSh, BWh , Csa, Csb	*diversipilis*	Gnathosoma: palpal setae *dF* serrate, setae *dG* filiform and smooth. Idiosoma wider than long. Propodonotal shield well outlined, with minute punctuation in medial part, very slightly concave anteriorly and posteriorly, with 6 pairs of slightly serrate setae. Laterally to propodonotal shield, small platelets with eye and one pair of serrate setae present. Venter. Anterio-medial part with 4 rows (11–14 setae) of plumose antero-median short setae; below, in posterior half of idiosoma, several rows of slightly serrate, thicker tapered setae. Coxal setae in formula: 2–2–4–3. Genital area with 4 genital setae *g1–g4* and 3 pairs of pseudanal setae *ps1–ps3*.	this study
G. inermis sp. nov.	*Ptyodactylus puiseuxi* Boutan, 1893	Phyllodactylidae	Israel	Csa, Csb	*latasti*	Gnathosoma: palpal setae *dF* filiform smooth or with barely discernible serration, setae *dG* filiform and smooth. Idiosoma rounded. Propodonotal shield smooth and well outlined, slightly concave in anterior and posterior part, with 13 pairs of very slightly plumose thick and blunt-pointed setae. Eyes absent. Venter. Anterior part with 1–2 rows of filiform smooth setae and 4 rows of slightly plumose thicker and tapered setae. Anteromedial part with plumose setae. Posterior part with lanceolate setae. Coxal setae in formula: 2–2–4–3. Genital area with 4 genital setae *g1–g4* and 11 pairs of pseudanal setae *ps1–ps11*.	this study

*present-day Central Asia, probably southern Kazakhstan or Turkmenistan ** ungrouped (due to unique leg-chaetotaxy pattern the species is not assigned to any of the established groups).

References for Supplementary Materials:

1. Bertrand, M.; Pfliegler, W.P.; Sciberras, A. Does the African native host explain the African origin of the parasite? The Maltese *Geckobia estherae* n. sp. parasitic on *Tarentola auritanica* (Acari: Raphignathoidea: Pterygosomatidae). *Acarologia* **2012**, 52(4), 353–366. https://doi.org/10.1051/acarologia/20122073
2. Trägårdh, I. Acariden aus Ägypten und dem Sudan. In *Results of the Swedish Zoological Expedition to Egypt and the White Nile. Part II*; Jägerskiöld, L.A., Ed.; Uppsala University: Uppsala, Sweden, **1905**; pp. 1–124.
3. Hirst, A.S. On the Parasitic Mites of the Suborder *Prostigmata* (Trombidioidea) Found on Lizards. *Journal of the Linnean Society of London, Zoology* **1926**, *36*, 173–200.
4. Jabbarpour, S. Türkiye’de Dağılış Gösteren Bazı Kertenkelelerde Ektoparazit Yaşayan Akarlar (Arachnida: Acarina). Ph.D. Thesis, Ege University, İzmir, Türkiye, 2016.
5. Eren, G.; Açıcı, M. An updated checklist of ticks and mites (Acari) reported on reptiles of Türkiye: New records and new host–parasite associations. *Acarological Studies* **2025**, 7(1), 12–41. https://doi.org/10.47121/acarolstud.1554694
6. Bertrand, M.; Paperna, I.; Finkelman, S. Pterygosomatidae: descriptions et observations sur les genres *Pterygosoma, Geckobia, Zonurobia* et *Hirstiella* (Acari: Actinedida). *Acarologia* **1999**, *40*, 275–304.
7. Bertrand, M.; Kukushkin, O.; Pogrebnyak, S. (2013). A New Species of Mites of the Genus *Geckobia* (Prostigmata, Pterygosomatidae), Parasitic on *Mediodactylus kotschyi* (Reptilia, Gekkota) from Crimea. *Vestnik Zoologii*, **47**(2), 99–111. https://doi.org/10.2478/vzoo-2013-0009

8. Fajfer, M. Two new mite species of the genus *Geckobia* Mégnin (Acariformes: Pterygosomatidae) from Israel. *Zootaxa* **2023**, *5227*, 251–264. https://doi.org/10.11646/zootaxa.5227.2.5
